# Antimicrobial and Antioxidant Activities and Effect of 1-Hexadecene Addition on Palmarumycin C_2_ and C_3_ Yields in Liquid Culture of Endophytic Fungus *Berkleasmium* sp. Dzf12 

**DOI:** 10.3390/molecules181215587

**Published:** 2013-12-13

**Authors:** Yan Mou, Jiajia Meng, Xiaoxiang Fu, Xiaohan Wang, Jin Tian, Mingan Wang, Youliang Peng, Ligang Zhou

**Affiliations:** 1 MOA Key Laboratory of Plant Pathology, Department of Plant Pathology, College of Agronomy and Biotechnology, China Agricultural University, Beijing 100193, China; E-Mails: muyan01987@163.com (Y.M.); mengjiajiax@163.com (J.M.); fuxiaoxiang123@qq.com (X.F.); 921439745@qq.com (X.W.); pstianjin@126.com (J.T.); pengyl@cau.edu.cn (Y.P.); 2 Department of Applied Chemistry, College of Science, China Agricultural University, Beijing 100193, China; E-Mail: wangma@cau.edu.cn

**Keywords:** spirobisnaphthalenes, palmarumycin C_2_, palmarumycin C_3_, endophytic fungus, *Berkleasmium* sp. Dzf12, 1-hexadecene, biological activity

## Abstract

Two spirobisnaphthalenes, namely palmarumycins C_2_ and C_3_, were isolated from cultures of the endophytic fungus *Berkleasmium* sp. Dzf12 after treatment with 1-hexadecene. After addition of 1-hexadecene at 10% to the medium on day 6 of culture, the maximal yields of palmarumycins C_2_ and C_3_ were obtained as 0.40 g/L and 1.19 g/L, which were 40.00 fold and 59.50 fold higher, respectively, in comparison with those of the control (0.01 g/L and 0.02 g/L). The results indicated that addition of 1-hexadecene can be an effective strategy for enhancing the production of palmarumycins C_2_ and C_3_ in liquid culture of endophytic fungus *Berkleasmium* sp. Dzf12. Palmarumycin C_3_ exhibited stronger antimicrobial and antioxidant activities than palmarumycin C_2_.

## 1. Introduction

Spirobisnaphthalenes (also namely bisnaphthospiroketals) are a growing group of fungal metabolites which contain two 1,8-dihydroxynaphthalene-derived units bridged through a spiroketal linkage [1,2]. They are divided into four subclasses, namely spiroxin-, preussomerin-, deoxypressomerin- and urnucratin-type spirobisnaphthalenes, according to their structural features [2,3]. Spirobisnaphthalenes possess a wide range of biological activities that suggest their potential applications in agriculture, medicine and the food industry [1,2,3,4].

In our previous studies, a variety of bioactive deoxypressomerin-type spirobisnaphthalenes were isolated from the endophytic fungus *Berkleasmium* sp. Dzf12 derived from a medicinal plant *Dioscorea zingiberensis* [5,6]. *Berkleasmium* sp. Dzf12 was also found to be a high producer of palmarumycins C_12_ and C_13_ [7,8,9,10,11]. As part of our ongoing program to search for other palmarumycins from *Berkleasmium* sp. Dzf12, both palmarumycins C_2_ and C_3_ were found to be the main spirobisnaphthalenes produced after addition of 1-hexadecene to the culture medium. In this report, we describe the isolation and structural elucidation of palmarumycins C_2_ and C_3_ along with their antimicrobial and antioxidant activities. The enhancing effects of 1-hexadecene on production of palmarumycins C_2_ and C_3_ in liquid culture of endophytic fungus *Berkleasmium* sp.Dzf12 were also studied.

## 2. Results and Discussion

### 2.1. HPLC Analysis of the Cultures Treated with 1-Hexadecene

**Figure 1:**
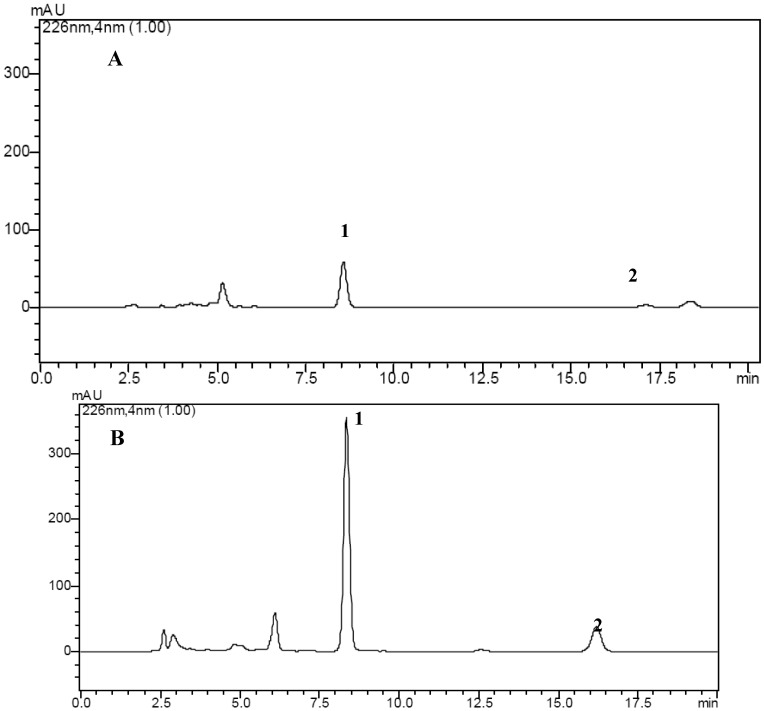
HPLC chromatograms of the crude ethyl acetate extracts from endophytic fungus *Berkleasmium* sp. Dzf12 untreated (**A**) and treated (**B**) with 1-hexadecene.

The crude ethyl acetate extract of the cultures of endophytic fungus *Berkleasmium* sp. Dzf12 treated with 1-hexadecene at 10% on day 3 of culture was analyzed by HPLC (Figure 1). Production of two main spirobisnaphthalene peaks (*i.e*., peaks 1 and 2, with retention times of 9.01 min and 15.93 min, respectively) was obviously promoted in the 1-hexadecene treated sample (Figure 1B).

### 2.2. Structural Identification of Two Main Spirobisnaphthalenes

**Figure 2:**
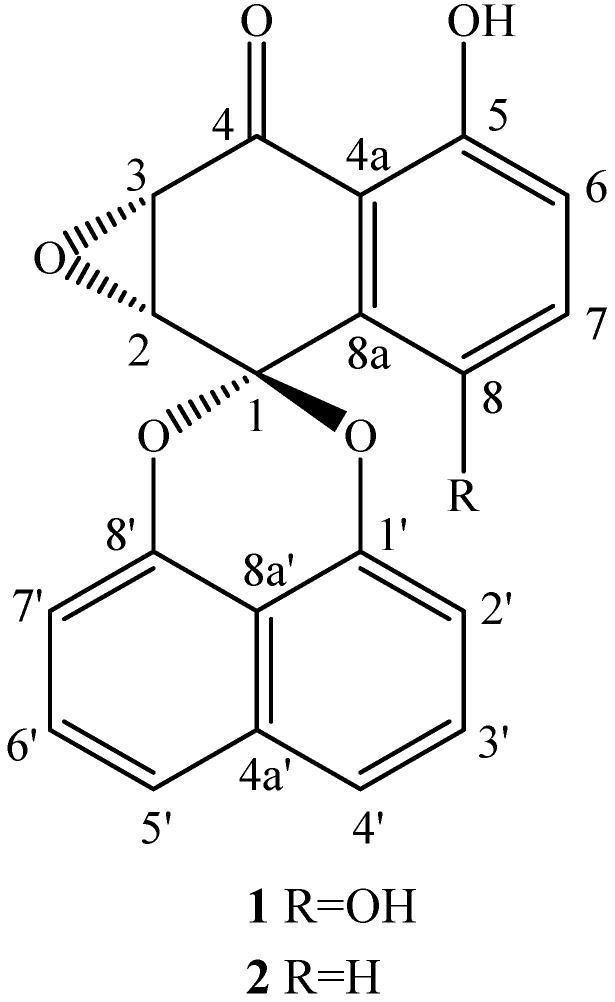
Chemical structures of palmarumycins C_3_ (**1**) and C_2_ (**2**).

An ethyl acetate extract (4.68 g) prepared from the cultures of endophytic fungus *Berkleasmium* sp. Dzf12 treated with 1-hexadecene, was subjected to column chromatography on silica gel, and further purified by semi-preparative HPLC to afford compounds **1** (49.0 mg) and **2** (15.4 mg). Their structures were elucidated based on the physicochemical and spectrometric data, and comparison of the properties and spectral characteristics with those described in the literature [12,13,14]. Both compounds were known spirobisnaphthalenes and confirmed as palmarumycin C_3_ (**1**) and palmarumycin C_2_ (**2**), respectively (Figure 2). Both palmarumycins C_3_ and C_2_ have been isolated from the fungus *Coniothyrium* sp. [12]. Palmarumycin C_2_ (also known as deoxypressomerin A) has also been isolated from other fungi such as *Microsphaeropsis* sp. BCC 3050, from one lichen *Dirinaria applanata* [15], and one unidentified fungus MF5916 from the dung of pig (*Sus scrofa*) [16].

### 2.3. Antimicrobial Activity of Palmarumycins C_2_ and C_3_

Antimicrobial activity of palmarumycins C_3_ (**1**) and C_2_ (**2**) was determined by their minimum inhibitory concentration (MIC) and median inhibitory concentration (IC_50_) values shown in Table 1. Both palmarumycin C_3_ (**1**) and C_2_ (**2**) exhibited good antibacterial activities on the six tested bacteria in comparison with the positive control streptomycin sulfate. Palmarumycin C_3_ (**1**) generally displayed stronger antimicrobial activity toward *A. tumefaciens*, *B. subtilis*, *P. lachrymans*, *R. solanacearum*, *S. haemolyticus*, *X. vesicatoria* and *M. oryzae* than palmarumycin C_2_ (**2**) except for the Gram-negative bacterium *S. haemolyticus*. Furthermore, palmarumycins C_3_ and C_2_ showed relatively weak inhibitory activity on spore germination of *M. oryzae* in comparison with their antibacterial activity. By comparing the structures (Figure 1) of palmarymycins C_3_ (**1**) and C_2_ (**2**), it can be speculated that the hydroxyl group at the C-8 position is favorable to the antimicrobial activity of palmarumycin C_3_.

**Table 1:** Antimicrobial activity of palmarumycins C_3_ (**1**) and C_2_ (**2**).

Microorganism	Palmarumycin C_3_ (1)		Palmarumycin C_2_ (2)		CK^+^	
	MIC (μg/mL)	IC_50 _(μg/mL)	MIC (μg/mL)	IC_50 _(μg/mL)	MIC (μg/mL)	IC_50 _(μg/mL)
*A. tumefaciens* ATCC 11158	6.25	2.59	6.25	5.16	25.00	11.96
*B. subtilis* ATCC 11562	6.25	3.22	6.25	7.12	37.50	20.58
*P. lachrymans* ATCC 11921	12.50	6.81	50.00	23.75	12.50	4.69
*R. solanacearum* ATCC 11696	12.50	5.69	25.00	17.48	50.00	41.26
*S. haemolyticus* ATCC 29970	6.25	5.74	6.25	2.13	12.50	4.24
*X. vesicatoria* ATCC 11633	6.25	5.85	12.50	9.92	50.00	22.83
*M. oryzae* P131	-	29.23	-	65.61	-	6.25

Note: MIC, minimum inhibitory concentration; IC_50_, median inhibitory concentration; -, not detected; CK^+^, positive controls for bacteria and fungus (*M. oryzae*) were streptomycin sulfate and carbendazim, respectively.

### 2.4. Antioxidant Activity of Palmarumycins C_2_ and C_3_

The antioxidant activity of palmarumycins C_3_ (**1**) and C_2_ (**2**) was assessed by 2,2'-diphenyl-1-picrylhydrazyl (DPPH) radical scavenging, β-carotene/linoleic acid bleaching capacity, and Fe^3+^ reducing power assays. For the DPPH radical scavenging and β-carotene/linoleic acid oxidation capacity tests, the lower IC_50_ values indicate higher antioxidant activity. As summarised in Table 2, palmarumyicn C_3_ (**1**) showed stronger antioxidant activity than palmarumycin C_2_ (**2**), but both were less active than the positive control dibutylhydroxytoluene (BHT).

**Table 2:** Antioxidant activity of palmarumycins C_3_ (**1**) and C_2_ (**2**).

Compound	IC_50_ (μg/mL)	
	DPPH Radical Scavenging	β-Carotene/Linoleic Acid
**Palmarumycin C_3_ (1)**	37.57	7.41
**Palmarumycin C_2_ (2)**	-	33.9
**BHT (CK^+^)**	19.15	2.35

Note: IC_50_, median inhibitory concentration; -, not detected.

**Figure 3:**
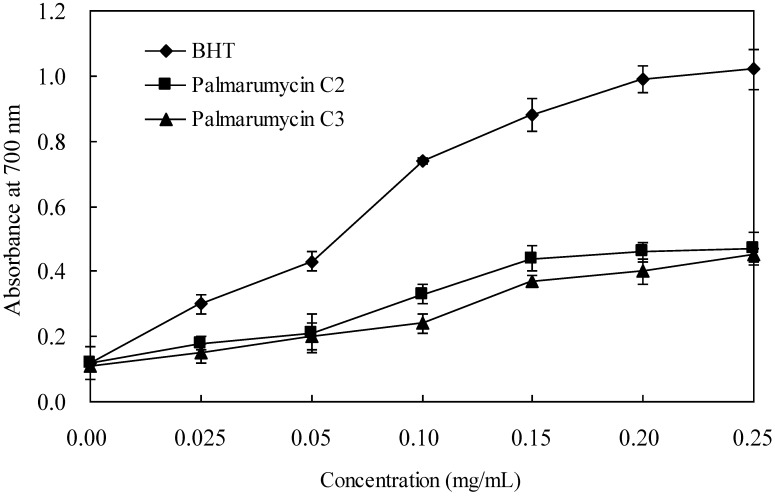
Reducing power of palmarumycins C_3_ (**1**) and C_2_ (**2**). Each value was expressed as a mean ± SD (*n* = 3).

For the reducing power assay, the presence of the antioxidant results in the reduction of the Fe^3+^/ferricyanide complex to the Fe^2+^ form. The amount of Fe^2+^ was then detected by measuring the formation of Perl’s Prussian blue at 700 nm [17]. As shown in Figure 3, a higher absorbance value indicates a stronger reducing power. Both palmarumycins C_3_ (**1**) and C_2_ (**2**) showed similar reducing power, which was weaker than that of the positive control, BHT.

### 2.5. Effects of 1-Hexadecene on Production of Palmarumycins C_2_ and C_3_

**Figure 4:**
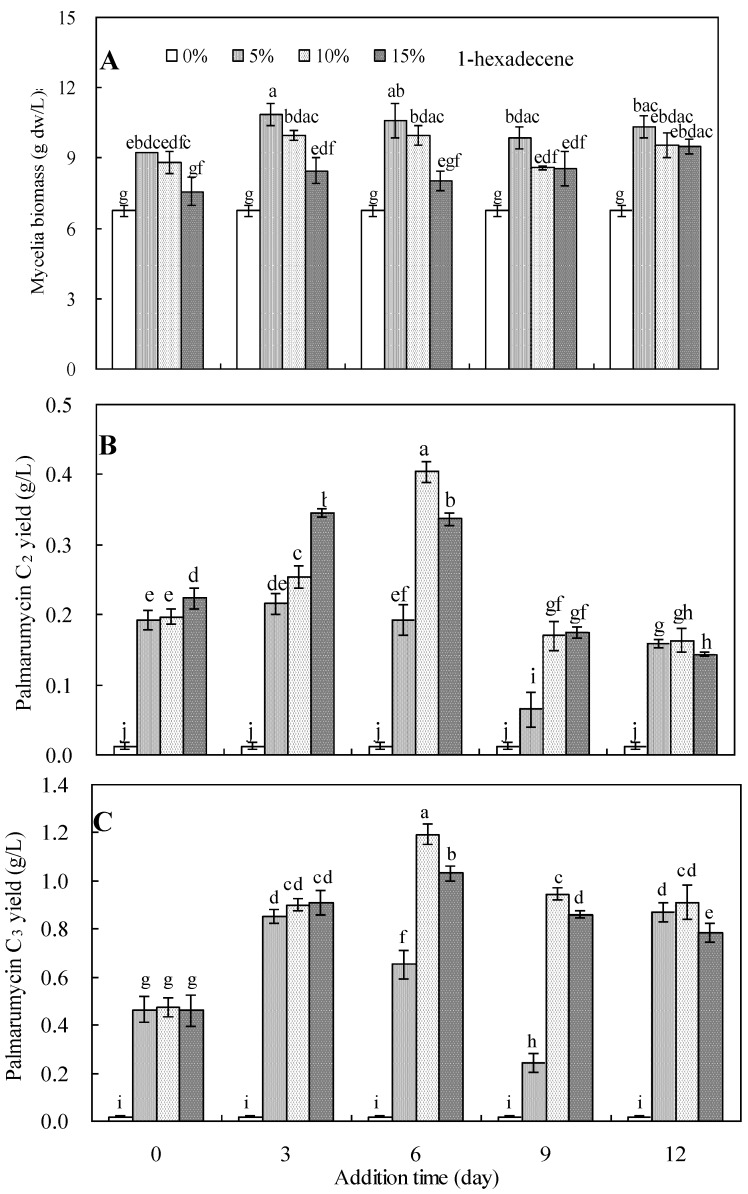
Effects of 1-hexadecene and its addition time on mycelia biomass (**A**), palmarumycin C_2_ (**B**) and palmarumycin C_3_ (**C**) production in liquid culture of *Berkleasmium* sp. Dzf12. 1-Hexadecene was applied at 5%, 10% and 15% on days 0, 3, 6, 9, and 12 of culture, respectively. The error bars represent standard deviations from three independent samples. Different letters indicate significant differences among the treatments at *p* = 0.05 level.

The effects of 1-hexadecene and its addition time on mycelia growth and palmarumycin production in *Berkleasmium* sp. Dzf12 liquid culture are presented in Figure 4. 1-Hexadecene significantly affected mycelia growth, especially at a concentration of 5% (Figure 4A). When 1-hexadecene was applied to the medium at 5% on day 3 and day 6 of culture, the mycelia biomass reached 10.87 and 10.60 g dw/L, respectively, which was about 1.60 fold of the control (6.56 g dw/L).

Hexadecene also obviously affected the yields of palmarumycins C_2_ and C_3_ in *Berkleasmium* sp. Dzf12 liquid culture (Figure 4B,C). Upon addition of 1-hexadecene at 10% on day 6 of culture, the maximal yields of palmarumycins C_2_ and C_3_ were obtained as 0.40 g/L and 1.19 g/L, which were 40.00 fold and 59.50 fold in comparison with those of the control (0.01g/L and 0.02 g/L, respectively). Thus, 1-hexadecene addition should be viewed as an effective strategy for palmarumycin C_2_ and C_3_ production in liquid culture of the endophytic fungus *Berkleasmium* sp. Dzf12.

## 3. Experimental

### 3.1. General

Melting points (uncorrected) of the compounds were measured on an XT4-100B microscopic melting-point apparatus (Tianjin Tianguang Optical Instruments Company, Tianjin, China). UV spectra were recorded with a TU-1810 UV-VIS spectrophotometer (Beijing Purkinje General Instrument Company, Beijing, China). HR-ESIMS was obtained on a Bruker Esquire 6000 LC/MS spectrometer and a Bruker Apex IV FTMS spectrometer (Bruker Daltonics Inc., Bremen, Germany). ^1^H and ^13^C NMR spectra were recorded at 400 and 100 MHz, respectively, on a Bruker Avance DRX-400 spectrometer in CDCl_3_ or acetone-*d*_6_, and the chemical shifts (δ) were reported in parts per million (ppm) relative to tetramethylsilane (TMS, δ = 0) as an internal standard.

The analytical HPLC was performed on two LC-20AT solvent delivery units, an SIL-20A autosampler, an SPD-M20A photodiode array detector, and CBM-20Alite system controller (Shimadzu, Kyoto, Japan), and a reversed-phase Ultimate TM XB C_18_ column (4.6 × 250 mm, 5 µm, Welch Materials, Inc., Ellicott, MD, USA). The HPLC column was isocratically eluted with MeOH–H_2_O (70:30, v/v) in 20 min at a flow rate of 1.0 mL/min. The temperature was maintained at 40 °C, and UV detection was at 226 nm. The sample injection volume was 10 µL. The LC solution multi-PDA workstation was employed to acquire and process chromatographic data.

Semi-preparative HPLC consisted of a K-501 pump, a K-2501 UV detector (Knauer, Berlin, Germany), a 2-mL sample loop, a workstation (Lumtech, Beijing, China), and a revesed-phase Luna C_18_ column (10 × 250 mm, 5 µm, Phenomenex, Torrance, CA, USA). The semi-preparative HPLC column was isocratically eluted with MeOH–H_2_O (70:30, v/v) in 40 min at a flow rate of 3.0 mL/min and UV detection at 226 nm. The sample injection volume was 2.0 mL.

### 3.2. Endophytic Fungus and Culture Condtions

The endophytic fungus *Berkleasmium* sp. Dzf12 (GenBank accession number EU543255) was isolated from the healthy rhizomes of the medicinal plant *Dioscorea zingiberensis* C. H. Wright (Dioscoreaceae) as described previously [5,18]. The living culture was maintained on potato-dextrose agar (PDA) slants and stored at 4 °C in the Herbarium of the College of Agronomy and Biotechnology, China Agricultural University.

For preparation of the inoculum, the mycelia of endophytic fungus Dzf12 from the solid stock culture were transferred into each 300-mL Erlenmeyer flask containing 100 mL of potato-dextrose broth (PDB) incubating for 4 days by shaking upon which the inoculum cultures at a ratio of 3% were transplanted to the fresh medium (each 150-mL Erlenmeyer flask filled with 30 mL medium), which composed of glucose (40 g/L), peptone (10 g/L), KH_2_PO_4_ (1.0 g/L), MgSO_4_·7H_2_O (0.5 g/L) and FeSO_4_·7H_2_O (0.05 g/L), and pH value was adjusted at 6.5. The fermentation cultures were incubated for 13 days on a rotary shaker in darkness at 150 rpm and 25 °C.

### 3.3. Application of 1-Hexadecene

1-Hexadecene (94% purity) was purchased from Alfa Aesar (a branch of Johnson Matthey Company, London, UK). 1-Hexadecene was filter-sterilized through a membrane (pore size, 0.45 µm), and then added to the 3-day-old mycelia cultures at concentration of 10%, and cultured for another 10 days. For investigating the effects of 1-hexadecene on production of palmarumycins C_2_ and C_3_, 1-hexadecene was individually added to the mycelia cultures at concentrations of 5%, 10% and 15% on days 0, 3, 6, 9, and 12 of culture. The parallel controls were conducted as an incubation of the fungus under identical conditions but without 1-hexadecene, and another incubation of 1-hexadecene without the fungus under the same conditions.

### 3.4. Extraction, Purification and Identification of Palmarumycins C_2_ and C_3_

A total of 2 L fermentation broth of *Berkleasmium* sp. Dzf12 was harvested after 13 days’ culture. The mycelia were separated from the culture filtrate by filtration, dried and powdered, and then extracted with methanol for three times. The concentrated methanol extract was suspended in water and extracted with ethyl acetate for three times. The culture filtrate was concentrated and extracted with ethyl acetate for three times. All the ethyl acetate extractions were combined and concentrated under vacuum at 35 °C on a rotary evaporator to obtain a total of 4.68 g crude extract.

The ethyl acetate extract was fractionated by silica gel column chromatography and eluted with a gradient of petroleum ether–acetone (from 100:0 to 0:100, v/v) to afford fractions A to K. Both fraction B (eluted with petroleum ether–acetone = 100:0.5, v/v) and fraction C (eluted with petroleum ether–acetone = 100:1.0, v/v) were further purified by semi-preparative HPLC to afford compound **1** (49.0 mg) and **2** (15.4 mg), respectively. The structures of the purified compounds were identified by HR-ESIMS, ^1^H- and ^13^C-NMR, by comparing their physicochemical and spectroscopic data with those of literatures [12,13,14]. The physicochemical and spectrometric data of two compounds are given as follows:

*Palmarumycin C_3_* (**1**). Yellow needles (acetone) with m.p. as 214–215 °C; UV (MeOH) λ_max_ 226, 275, 297 and 327 nm. Its molecular formula of C_20_H_12_O_6_ was determined by HR-ESIMS *m/z* 349.0703 ([M + H]^+^, calcd. for C_20_H_13_O_6_, 349.0707), 371.0522 ([M + Na]^+^, calcd for C_20_H_12_O_6_Na, 371.0526). ^1^H- NMR (400 MHz, CDCl_3_) δ: 11.45 (1H, s, OH-5), 7.66 (1H, d, *J* = 8.4 Hz, H-4'), 7.60 (1H, d, *J* = 8.4 Hz, H-5'), 7.54 (1H, d, *J* = 7.8 Hz, H-3'), 7.50 (1H, d, *J* = 8.2 Hz, H-6'), 7.35 (1H, s, OH-8), 7.27 (1H, d, *J* = 9.3 Hz, H-7), 7.24 (1H, d, *J* = 7.6 Hz, H-2'), 7.09 (1H, d, *J* = 9.2 Hz, H-6), 7.03 (1H, d, *J* = 7.5 Hz, H-7'), 4.02 (1H, d, *J* = 4.0 Hz, H-3), 3.62 (1H, d, *J* = 4.0 Hz, H-2); ^13^C-NMR (100 MHz, CDCl_3_) δ: 99.1 (s, C-1), 53.3 (d, C-2), 52.8 (d, C-3), 195.8 (s, C-4), 111.3 (s, C-4a), 157.2 (s, C-5), 122.6 (d, C-6), 130.0 (d, C-7), 149.4 (s, C-8),. 116.2 (s, C-8a), 145.3 (s, C-1’), 111.1 (d, C-2'), 127.8 (d, C-3'), 122.9 (d, C-4'), 134.3 (s, C-4a'), 121.5 (d, C-5'), 128.0 (d, C-6'), 110.5 (d, C-7'), 146.6 (s, C-8'), 113.2 (s, C-8a'). The structure (Figure 2) was confirmed by comparison with the literature data [12,13,14].

*Palmarumycin C_2_* (**2**). Pale yellow needles (acetone) with m.p. as 222–224 °C; UV (MeOH) λ_max_ as 226, 267, 299 and 327 nm. Its molecular formula of C_20_H_12_O_5_ was determined by HR-ESIMS *m/z* 333.0758 ([M + H]^+^, calcd. for C_20_H_13_O_5_, 333.0758). ^1^H-NMR (400 MHz, acetone-*d*_6_) δ: 11.33 (1H, s, OH-5), 7.78 [1H, pseudo-t (dd), *J* = 8.1 and 8.1 Hz, H-7], 7.70 (1H, d, *J* = 8.3 Hz, H-4'), 7.66 (1H, d, *J* = 8.2 Hz, H-5'), 7.61 [1H, pseudo-t (dd), *J* = 7.7 Hz and 8.2 Hz, H-3'), 7.53 [1H, pseudo-t (dd), J = 7.8 and 8.1 Hz, H-6'], 7.46 (1H, d, *J* = 7.6 Hz, H-8), 7.21 (1H, d, *J* = 7.5 Hz, H-2'), 7.18 (1H, d, *J* = 8.5 Hz, H-6), 6.98 (1H, d, *J* = 7.5 Hz, H-7'), 4.26 (1H, d, *J* = 4.0 Hz, H-3), 3.84 (1H, d, *J* = 4.0 Hz, H-2); ^13^C-NMR (100 MHz, CDCl_3_) δ: 96.1 (s, C-1), 53.3 (d, C-2, C-3), 196.6 (s, C-4), 112.4 (s, C-4a), 162.0 (s, C-5), 120.2 (d, C-6), 137.8 (d, C-7), 119.2 (d, C-8), 137.0 (s, C-8a), 146.8 (s, C-1'), 110.4 (d, C-2'), 127.9 (d, C-3'), 122.0 (d, C-4'), 134.3 (s, C-4a'), 121.5 (d, C-5'), 127.7 (d, C-6'), 109.4 (d, C-7'), 147.0 (s, C-8'), 112.9 (s, C-8a'). The structure (Figure 2) was confirmed by comparison with the literature data [12,13,14].

### 3.5. Antimicrobial Activity Assay

Two Gram-positive (*Bacillus subtilis* ATCC 11562 and *Staphylococcus haemolyticus* ATCC 29970) and four Gram-negative (*Agrobacterium tumefaciens* ATCC 11158, *Pseudomonas lachrymans* ATCC 11921, *Ralstonia solanacearum* ATCC 11696 and *Xanthomonas vesicatoria* ATCC 11633) bacteria were selected for antibacterial activity assay by using the chromogenic reagent 3-(4,5-dimethylthiazol-2-yl)-2,5-diphenyl tetrazolium bromide (MTT) [19,20]. Streptomycin sulfate was used as the positive control, and 30% DMSO was used as the negative control. The minimum inhibitory concentration (MIC) was defined as the lowest concentration of the sample that resulted in visible growth inhibition of the microorganisms. The median inhibitory concentration (IC_50_) was calculated using the linear relation between the inhibitory probability and concentration logarithm [21].

The spore germination assay using the rice blast fungus *Magnaporthe oryzae* (strain P131) was employed to detect the antifungal activity of the compounds [22]. The positive control was carbendazim, and the negative control was 30% acetone. The IC_50_ value calculation for the spore germination inhibition was the same as that for antibacterial activity assay. All tests were performed in triplicate.

### 3.6. Antioxidant Activity Assay

Antioxidant activity of palmarumycins C_3_ (**1**) and C_2_ (**2**) was determined by 2,2'-diphenyl-1-picrylhydrazyl (DPPH) radical scavenging, β-carotene-linoleic acid bleaching, and Fe^3+^ reducing power assays.

The DPPH radical scavenging assay was performed according to the method of Qiao *et al.* [23] with slight modifications. 0.2 mM DPPH methanol solution (80 µL) was mixed with the purified compound in methanol (20 µL) in a 96-well microplate. The mixture was shaken vigorously and allowed to reach a steady state at 37 °C for 30 min. The absorbance of solution was measured at 517 nm using a microplate spectrophotometer (PowerWave HT, BioTek Instruments, Winooski, VT, USA). Butylated hydroxytoluene (BHT) was used as a reference. The DPPH radical scavenging activity was calculated according to the following equation:

Scavenging rate (%) = (1 − *A*s/*A*c) × 100

where *A*c was the absorbance of pure DPPH in methanol, *A*s was the absorbance of DPPH in the presence of sample. The IC_50_ value was calculated using the linear relation between the scavenging rate and logarithm of concentration. All tests were performed in triplicate.

The β-carotene/linoleic acid oxidation capacity of the compounds was measured according to Dapkevicius *et al.* [24] with slight modifications. β-Carotene (0.5 mg) dissolved in CHCl_3_ (3 mL) and then linoleic acid (25 µL) and Tween-40 (200 mg) were added and mixed adequately. The mixture was concentrated under vacuum at 30 °C. Distilled water (50 mL) was then added. The reaction mixture was shaken at room temperature for 30 min. The mixtures of β-carotene/linoleic acid solution (90 µL) and the test sample solution (10 µL) at various concentrations were incubated at 50 °C for 2 h, and the absorbance was recorded at 460 nm against the blank. The negative control was distilled water (10 µL), and BHT at the same concentrations was used as the positive control. The level of antioxidation was calculated according to the following equation:

Antioxidation effect (%) = (*A*s/*A*c) × 100

where *A*c was the absorbance before the reaction of β-carotene/linoleic acid with the sample, and *A*s showed the absorbance after 2 h reaction of β-carotene/linoleic acid with the sample. The IC_50_ value was calculated using the linear relation between the antioxidation effect and concentration logarithm. All tests were performed in triplicate.

The Fe^3+^ reducing power of the sample was employed according to the method of Oyaizu *et al.* [25] with slight modifications. At various concentrations, sample (50 µL) was mixed with phosphate buffer (30 µL, 0.2 M, pH 6.6) and potassium ferricyanide (20 µL, 1%, w/v). The mixture was incubated for 20 min at 50 °C. After incubation, trichloroacetic acid (TCA, 70 µL, 10%, w/v) and FeCl_3_ (10 µL, 1%, w/v) were added and maintained for 10 min. The absorbance at 700 nm was measured as the reducing power. Increased absorbance of the reaction mixture indicated the increased reducing power of the sample. BHT was used as the positive control. All tests were performed in triplicate.

### 3.7. Determination of Mycelia Biomass and Quantificaiton of Palmarumycins C_2_ and C_3_

To measure the effects of 1-hexadecene on the *Berkleasmium* sp. Dzf12 mycelia biomass and palmarumycin production, the mycelia were obtained from the liquid medium by filtration under vacuum, and then dried at 50–55 °C in an oven to a constant dry weight (dw). Palmarumycin extraction and quantification were carried out as previously described [10]. Briefly, the dried mycelia were ground into powder, and then mycelia (50 mg) was extracted with methanol-chloroform (5 mL, 9:1, v/v) under sonication for 60 min. Through filtration, the filtrate was evaporated to dryness and re-dissolved in methanol (1 mL) for HPLC analysis.

For quantitative analysis of palmarumycins C_2_ and C_3_ in the culture filtrate, culture filtrate without mycelia (30 mL) was fractionated with the same volume of ethyl acetate three times. The combined ethyl acetate solution was concentrated and re-dissolved in methanol (1 mL) for HPLC analysis.

The purified palmarumycins C_2_ and C_3_ were used as the standards. The linear equation of palmarumycin C_2_ (**2**) by HPLC analysis was *Y* = 6378070*X* − 59377.78 (*R*^2^ = 0.9999), and that of palmarumycin C_3_ (**1**) was *Y* = 26716350*X* − 101002.6 (*R*^2^ = 0.9999), where *Y* was the peak area, *X* was quality (µg) of the sample injected for each time, and *R* was the correlation coefficient.

### 3.8. Statistical Analysis

All tests were carried out in triplicate, and the results were represented by their mean values and the standard deviations (SD). The data were submitted to analysis of variance (one-way ANOVA) to detect significant differences by PROC ANOVA of SAS version 8.2. The term significant has been used to denote the differences for which *p* ≤ 0.05.

## 4. Conclusions

In this work, two spirobisnaphthalenes (palmarumycins C_2_ and C_3_) were first isolated from the liquid cultures of endophytic fungus *Berkleasmium* sp. Dzf12 treated with 1-hexadecene. Other peaks with retention times ranging from 2.5 min to 7.5 min also appeared in the sample treated with 1-hexadecene (Figure 1). These compounds should be further studied. After addition of 1-hexadecene to the medium at 10% on day 6 of culture, the maximal yields of palmarumycins C_2_ and C_3_ were obtained (0.40 g/L and 1.19 g/L), which were 40.00 fold and 59.50 fold higher in comparison with those of the control (0.01 g/L and 0.02 g/L, respectively). Addition of 1-hexadecene in liquid culture of endophytic fungus *Berkleasmium* sp. Dzf12 could be an efficient method to produce palmarumycins C_2_ and C_3_.

Some alkanes (*i.e.*, *n*-hexane, *n*-dodecane and *n*-hexadecane) were previously reported to increase metabolite production by acting as oxygen-vectors [26,27,28]. Typical examples included lycopene and β-carotene production, enhanced by both *n*-hexane and *n*-dodecane in *Blakeslea trispora* [26], lovastatin production enhanced by *n*-dodecane in *Aspergillus terreus* [27], and arachidonic acid production enhanced by *n*-hexadecane [28]. To our knowledge, this is the first report on the metabolite production enhanced by 1-hexadecene in microbial culture. 1-Hexadecene, belonging to the alkenes, probably either acts as the oxygen-vector or plays a role in the redox system to increase palmarumycin C_2_ and C_3_ production in *Berkleasmium* sp. Dzf12, which needs further investigation. Palmarumycins C_2_ and C_3_ were considered as the intermediates in the biosynthetic pathway of spirobisnaphthalenes [29]. It should be beneficial for us to further study the biosynthesis of spirobisnaphthalenes.

The previous results demonstrated that both palmarumycins C_2_ and C_3_ showed good antibacterial, antifungal and algicidal activities [12]. In addition, palmarumycin C_2_ exhibited inhibitory activity on Ras farnesyl-protein transferase (FPTase) with the IC_50_ value as 10 µM [16]. Other biological activities of palmarumycins C_2_ and C_3_ need to be further studied in detail.
